# Autoimmune Encephalitis and Other Neurological Syndromes With Rare Neuronal Surface Antibodies in Children: A Systematic Literature Review

**DOI:** 10.3389/fped.2022.866074

**Published:** 2022-04-20

**Authors:** Claudio Ancona, Valentina Masenello, Matteo Tinnirello, Luca Mattia Toscano, Andrea Leo, Chiara La Piana, Irene Toldo, Margherita Nosadini, Stefano Sartori

**Affiliations:** ^1^Department of Women's and Children's Health, University Hospital of Padova, Padova, Italy; ^2^Paediatric Neurology and Neurophysiology Unit, Department of Women's and Children's Health, University Hospital of Padova, Padova, Italy; ^3^Neuroimmunology Group, Paediatric Research Institute “Città della Speranza”, Padova, Italy

**Keywords:** neuronal surface antibody syndromes, autoimmune encephalitis, children, pediatrics, central nervous system

## Abstract

Neuronal surface antibody syndromes (NSAS) are an expanding group of autoimmune neurological diseases, whose most frequent clinical manifestation is autoimmune encephalitis (AE). Anti-NMDAR, anti-LGI1, and anti-CASPR2 autoimmunity represent the most described forms, while other NSAS are rarer and less well-characterized, especially in children. We carried out a systematic literature review of children with rare NSAS (with antibodies targeting D2R, GABAAR, GlyR, GABABR, AMPAR, amphiphysin, mGluR5, mGluR1, DPPX, IgLON5, and neurexin-3alpha) and available individual data, to contribute to improve their clinical characterization and identification of age-specific features. Ninety-four children were included in the review (47/94 female, age range 0.2–18 years). The most frequent NSAS were anti-D2R (28/94, 30%), anti-GABAAR (23/94, 24%), and anti-GlyR (22/94, 23%) autoimmunity. The most frequent clinical syndromes were AE, including limbic and basal ganglia encephalitis (57/94, 61%; GABAAR, D2R, GABABR, AMPAR, amphiphysin, and mGluR5), and isolated epileptic syndromes (15/94, 16%; GlyR, GABAAR). With the limitations imposed by the low number of cases, the main distinctive features of our pediatric literature cohort compared to the respective NSAS in adults included: absent/lower tumor association (exception made for anti-mGluR5 autoimmunity, and most evident in anti-amphiphysin autoimmunity); loss of female preponderance (AMPAR); relatively frequent association with preceding viral encephalitis (GABAAR, D2R). Moreover, while SPS and PERM are the most frequent syndromes in adult anti-GlyR and anti-amphiphysin autoimmunity, in children isolated epileptic syndromes and limbic encephalitis appear predominant, respectively. To our knowledge, this is the first systematic review on rare pediatric NSAS. An improved characterization may aid their recognition in children.

## Introduction

Neurological syndromes with neuronal surface antibodies (NSAbs) are an expanding group of conditions whose most frequent clinical manifestation is autoimmune encephalitis (AE). They are characterized by more recent identification, rarer tumor association, higher frequency in children, direct pathogenic role of autoantibodies, and therefore better immunotherapy response compared with syndromes with antibodies against intracellular neuronal antigens (1–9).

The most frequent and best characterized NSAS are anti-N-methyl-D-aspartate receptor (NMDAR) encephalitis (10–12), and autoimmunity with NSAbs targeting the leucine-rich, glioma-inactivated protein-1 (LGI1), and the contactin-associated protein-2 (CASPR2) (13–15). In children, myelin oligodendrocyte glycoprotein (MOG)-associated disorders (MOGAD) represent another major class of autoimmune disorders where MOG, a minor component of the central nervous system myelin sheath, is the target antigen; pediatric MOGAD most frequently manifest with acute demyelinating syndromes such as acute disseminated encephalomyelitis or optic neuritis, but rarer manifestations, including cortical encephalitis, have been described (16).

Rarer and less characterized NSAS, especially in children, include those with antibodies targeting the glycine receptor (GlyR) (17), the α-amino-3-hydroxy-5-methyl-4-isoxazolepropionic acid receptor (AMPAR) (18), the γ-aminobutyric acid-A and B receptor (GABAAR, GABABR) (19, 20), the metabotropic glutamate receptor 1 and 5 (mGluR1, mGluR5) (9, 21, 22), the dopamine-2 receptor (D2R) (23), the dipeptidyl-peptidase-like protein-6 (DPPX) (24), the immunoglobulin-like cell adhesion molecule 5 (IgLON5) (25), neurexin-3alpha (26), and amphiphysin (27).

We hereby carry out a systematic literature review of these latter rarer and less characterized NSAS in children, with the aim of contributing to their clinical description.

## Methods

We conducted a systematic literature review on rare NSAS in children (D2R, GABAAR, GlyR, GABABR, AMPAR, amphiphysin, mGluR5, mGluR1, DPPX, IgLON5, and neurexin-3alpha).

We searched Pubmed from inception up to 10 September 2021, with the following search keys: “((complete name of the receptor) or (abbreviation of the name)) and (autoimmune or antibod^*^ or abs) and (encephalit^*^ or syndrome).” We included published details of the patients with onset of neurological symptoms in pediatric age (0–18 years) with serum and/or cerebrospinal fluid (CSF) positivity for the searched antibodies, and available individual data (minimum data: age and clinical syndrome); large cohorts providing only pooled data were excluded. In the results section and in Table 1, the denominators may differ due to heterogeneous data availability.

**Table 1:** Most frequent features associated with rare NSAS in paediatric age according to our systematic literature review (antibodies targeting D2R, GABAAR, GlyR, GABABR, AMPAR, amphiphysin, mGluR5, mGluR1, DPPX, IgLON5, and neurexin-3alpha).

**Most frequent features in rare neuronal surface antibody syndromes (NSAS) in children**									
**Antibody target (n** **°** **of cases included in our literature review)**	**Sex** **Age range**	**Most frequent clinical syndromes**	**Tumour**	**Abnormal brain MRI**	**Abnormal EEG** **°**	**Abnormal CSF** ^**°°**^	**Rate of antibody detection in serum (S) and cerebrospinal fluid (CSF)**	**Immuno** **therapy (IT)**	**Days from onset of neurological symptoms to first IT**	**Outcome (relapse rate, mRS** ^**#**^ **, other data on outcome)**
D2R∧ (*n* = 28)	F: 54% (15/28) 0.8–17 y	Basal ganglia encephalitis: 71 (20/28) AE: 4% (1/28)	0% (0/28)	71% (15/21) Basal ganglia T2 hyperintensity	57% (12/21) Slowing of activity	50% (10/20)	S: 96.4% (27/28) CSF: 33.3% (2/6)* Double positives: 7% (2/28) (anti-NMDAR)	Any IT: 81% (17/21) IVMP: 62% (13/21) OP: 71% (15/21) IVIG: 43% (9/21) TPE: 5% (1/21) RTX: 5% (1/21)	Median 35 Mean 525 Range 2–2920 (d.a. 9/21)	Relapse: 43% (9/21) mRS: median 2, mean 1.5, range 0–5 (d.a. 21/21) Residual symptoms: Cognitive: 62% (13/21) Psychiatric: 42% (9/21) Movement disorder: 38% (8/21)
		Tourette's syndrome: 14% (4/28)		0% (0/4)	NA	NA		0% (0/4)	No IT	NA
		Isolated psychosis: 11% (3/28)		0% (0/3)	NA	NA		0% (0/3)	No IT	67% (2/3) unable to return to school or start employment
GABAAR (*n* = 23)	F: 57% (13/23) 0.2–17 y	AE with seizures: 78% (18/23) SPS: 4% (1/23) Right temporal lobe epilepsy with hippocampal sclerosis: 4% (1/23) Catatonia: 4% (1/23) West syndrome and Lennox-Gastaut syndrome: 4% (1/23) Psychiatric disorder: 4% (1/23)	4% (1/23) Hodgkin lymph.	70% (16/23) Increased T2/FLAIR signal with cortical-subcortical involvement, often multifocal, often involving the frontotemporal lobes Hippocampal sclerosis	100% (17/17) Generalized slowing and/or epileptiform activity	66.7% (12/18)	S: 100% (20/20) CSF: 75% (12/16)**	First-line IT: 78% (18/23) (IVMP, IVIG, TPE) Second-line IT or steroid sparers: 44% (11/23) (RTX, CPH, MMF)	Median 11 Mean 28.8 Range 2–91 (d.a. 4/23)	Relapse: 54% (7/13) mRS: median 4, mean 2.4, range 0–4 (d.a. 5/23) Full recovery: 37% (7/19) Partial recovery: 58% (11/19) Death: 4% (1/19)
GlyR (*n* = 22)	F: 55% (12/22) 0.8–17 y	Mesial temporal lobe epilepsy with hippocampal sclerosis: 23% (5/22) Other epilepsy syndromes, epileptic encephalopathy 36% (8/22) SPS: 23% (5/22) PERM: 9% (2/22) Transverse myelitis: 4.5% (1/22) ADEM with optic neuritis: 4.5% (1/22)	0% (0/23)	46% (6/13) Hippocampal sclerosis in patients with mesial temporal lobe epilepsy with hippocampal sclerosis	77% (10/13)	11% (1/9)	S: 82% (18/22) CSF: 86% (6/7)***	Any IT: 65% (13/20) CS: 40% (8/20) IVIG: 50% (10/20) TPE: 25% (5/20) RTX: 5% (1/20) AZA: 10% (2/20)	Median 45 Mean 344 Range 44–943 (d.a. 3/22)	Relapse: 14% (3/22) mRS: median 1, mean 1.2, range 0–3 (d.a. 9/22)
GABABR (*n* = 6)	F: 3/6 3–18 y	Limbic encephalitis: 5/6 AE: 1/6	0/6	2/3 T2/FLAIR hyperintensity of basal ganglia, cerebellum and brainstem involvement	1/2	2/2	S: 4/6 CSF: 4/4**** Double positives: 3/6 (serum anti-VGCC in 2/6), CSF anti-GABAAR in 1/6)	Any IT: 4/5 CS: 3/5 IVIG: 3/5 TPE: 2/5	NA	Relapse: 0/5 mRS: median 2.5, mean 2.5, range 1–4 (d.a. 2/6) Death: 1/6
AMPAR (*n* = 4)	F: 1/4 2–18 y	Limbic encephalopathy with cognitive-behavioural symptoms and movement disorders: 4/4	0/4	1/4 T2/FLAIR hyperintensities with contrast enhancement in bilateral cerebellar hemispheres	2/4	1/3 Lymphocytic pleocytosis	S: 3/4 CSF: 2/3	Any IT: 3/4 CS, IVIG: 2/4 RTX: 1/4	Median 83 Mean 591.5 Range 10–2190 (d.a. 4/4)	Relapse: 1/3 mRS: 0 (d.a. 1/4)
Amphiphysin (*n* = 4)	F: 1/4 9–12 y	Limbic encephalitis: 4/4	0/4	4/4 FLAIR signal abnormalities in mediotemporal area or T2 hyperintensity in the temporal lobes	1/1 Multifocal bilateral epileptiform discharges	2/4	S: 4/4 CSF: NA Double positives: 1/4 (anti-GAD)	Any IT: 3/4 IVMP: 2/4 OP: 1/4 IVIG:2/4	Median 61 Mean 1237.3 Range 1–3650 (d.a. 3/4)	Relapse: 0/4 mRS: median 3, mean 3, range 2–4 (d.a. 4/4)
mGluR5 (*n* = 4)	F: 2/4 6–16 y	Limbic encephalitis: 4/4	3/4 Hodgkin lymph.	2/4 Frontal, occipital lobes, and cerebellum involvement	3/4 Diffuse slowing of rhythms (3/4) Epileptiform discharges (2/4)	4/4 Pleocytosis (4/4) OCB (3/4)	S: 2/2 CSF: 4/4	Any IT: 3/4 CS: 3/4 IVIG: 2/4 TPE: 1/4 RTX: 1/4	NA	Relapse: 1/4 mRS: NA Full recovery: 2/4 Partial recovery: 2/4
mGluR1 (*n* = 2)	F: 0/2 3–6 y	Acute cerebellar syndrome: 2/2	0/2	1/2 Mild cerebellar oedema	NA	2/2 Pleocytosis and OCB	S: 0/1 CSF: 2/2	Any IT: 2/2 IVMP: 2/2 IVIG: 2/2	10 and 21	Relapse: 0/2 mRS: 1 (d.a. 1/2) Full recovery: 2/2
DPPX (*n* = 1)	M 15 y	PERM (hyperkplexia, cerebellar ataxia, nystagmus and stiffness)	No	Normal brain MRI	Normal	Pleocytosis	S: positive 1:10,000 CSF: positive 1:320	IVMP, IVIG, TPE (onset) RTX (at relapse)	365	Relapse: yes Gradual improvement
IgLon5 (*n* = 1)	M 2 y	Sleep and movement disorders (horizontal nystagmus, postural unsteadiness, and ankle stiffness)	Langerh. cell histiocyt.	Meningeal enhancement	Normal	Normal	S: positive 1:30 CSF: negative	IT (not otherwise specified)	61	NA
Neurexin-3α		No paediatric patients identified	/	/	/	/	/	/	/	/

*In this table and in the Results, denominators may differ due to heterogenous data availability*.

*ADEM, acute disseminated encephalomyelitis; AE, autoimmune encephalitis; AMPAR, α-amino-3-hydroxy-5-methyl-4-isoxazolepropionic acid receptor; AZA, azathioprine; CPH, cyclophosphamide, CS, corticosteroids; CSF, cerebrospinal fluid; d.a., data available; D2R, dopamine-2 receptor; DPPX, dipeptidyl-peptidase-like protein-6; EEG, electroencephalography; F, female; GABAAR and GABABR, γ-aminobutyric acid-A and B receptor; GlyR, glycine receptor; Hodgkin lymph., Hodgkin lymphoma; IgG, immunoglobulin; IgLON5, immunoglobulin-like cell adhesion molecule 5; IT, immunotherapy; IVIG, intravenous immunoglobulin; IVMP, intravenous methylprednisolone; Langer. cell histiocyt., Langerhans cell histiocytosis; MMF, mycophenolate mofetil; M, male; mo, month/months; mGluR1 and mGluR5, metabotropic glutamate receptor type 1 and 5; MRI, magnetic resonance imaging; mRS, modified Rankin Scale; NA, not available; OCB, oligoclonal bands; PERM, progressive encephalomyelitis with rigidity and myoclonus; RTX, rituximab; S, serum; SPS, stiff-person syndrome; TPE, therapeutic plasma exchange; y, year/years; VGCC, voltage-gated calcium channel*.

*°Abnormal EEG: slow or disorganised activity and/or epileptic activity*.

*^°°^Abnormal CSF: leukocytes > 4 μl^−1^ and/or proteins > 45 mg/dl and/or intrathecal IgG synthesis (OCB)*.

*^#^mRS was included as reported in the original articles*.

*∧ Despite exclusion from our literature review due to unavailability of individual data, patients with Sydenham's chorea and PANS/PANDAS have also been reported (23)*.

*Among anti-D2R patients in whom antibodies were tested both in serum and CSF (6/28, 21.4%), in 66.6% (4/6) antibodies were positive only in serum, in 16.6% (1/6) antibodies were positive only in CSF, and in 16.6% (1/6) in both CSF and serum.

**In 12 patients in whom anti-GABAAR antibodies were tested in both serum and CSF, serum and CSF positivity was reported in 75% (9/12), and in the remaining 25% (3/12) antibodies were positive in serum and negative in CSF.

***Among the seven anti-GlyR patients who tested both serum and CSF, antibodies were positive only in CSF in 57.1% (4/7), in both serum and CSF in 28.6% (2/7), and only in serum in 14.3% (1/7).

****Among anti-GABABR patients with paired serum and CSF samples, in 75% (3/4) antibodies were positive only in CSF, and in 25% (1/4) both in serum and CSF.

## Results

Our literature review disclosed 94 published cases meeting the inclusion criteria (Table 1, Supplementary Table 1).

### Anti-D2R Autoimmunity

#### Demographics

Twenty-eight children with anti-D2R autoimmunity were identified (23, 28–33) (15/28, 53.6% female). The median age at onset was 5.5 years (mean 5.4, range 0.8–17).

#### Clinical Syndromes and Symptoms

Four main clinical syndromes were identified, namely, basal ganglia encephalitis (20/28, 71.4%) (BGE), Tourette's syndrome (4/28, 14.3%), isolated psychosis (3/28, 10.7%), and AE (1/28, 3.6%). It should also be noted that despite exclusion from our literature review due to unavailability of individual data, patients with Sydenham's chorea and PANS/PANDAS have also been reported (23).

In BGE-AE patients (21/28), the most frequent subgroups of symptoms were movement disorders (20/21, 95.2%): dystonia, chorea, parkinsonism, ocular symptoms; psychiatric symptoms (15/21, 71.4%): agitation, personality change and psychosis; cognitive symptoms (15/21, 62.5%): cognitive decline, impaired executive functions, attention, fine motor coordination, memory; speech disorder (9/21, 42.9%); sleep disorders (5/21, 23.8%); cerebellar signs (5/21, 23.8%); seizures (4/21, 19%). Among patients with Tourette's syndrome, 4/4 had tics, and 2/4 had psychiatric disorders. Psychiatric disturbances were the only symptom in 3/3 in the isolated psychosis subgroup.

None of the patients had associated malignancies (0/28, 0%). Infections preceding the neurological onset were reported in 42.9% (9/21) of cases, including previous herpes simplex virus 1 (HSV1) encephalitis, or post-vaccine (2/21, 9.5%). In 1/4 patients with Tourette's syndrome a previous streptococcal infection was diagnosed and in 2/4 elevated antistreptolysin-O titer was detected. All 3/3 patients with isolated psychosis had past neuropsychiatric comorbidities and positive psychiatric family history.

#### Anti-D2R Antibodies

Antibodies were tested in serum in 100% (28/28) patients (positive in 27/28, 96.4%), and in CSF in 21.4% (6/28) (positive in 2/6, 33.3%). About 7.1% (2/28) of patients had coexistent anti-NMDAR antibodies. Antibody assay used was cell-based assay (CBA) in 26/28, and ELISA in 2/28 (31, 33).

#### Investigations

Among the BGE-AE subgroup, abnormal brain magnetic resonance imaging (MRI) was reported in 71.4% (15/21), mostly showing basal ganglia T2 hyperintensity, resolving during the recovery phase in 60% (9/15). Abnormal electroencephalography (EEG) was reported in 57.1% (12/21), showing non-specific slowing. CSF data was available in 95.2% (20/21) patients and disclosed abnormalities in 50% (10/20). No abnormalities on imaging (MRI or computerised tomography scan) were found among patients with Tourette's syndrome and with isolated psychosis. EEG and CSF were not performed in these latter two subgroups of patients.

#### Treatment

Immunotherapy was used in 89.9% (17/21) of BGE-AE patients: intravenous methylprednisolone (13/21, 61.9%) (IVMP), oral prednisone (15/21, 71.4%) (OP), intravenous immunoglobulin (9/21, 42.9%) (IVIG), therapeutic plasma exchange (1/21, 4.8%) (TPE), rituximab (1/21, 4.8%), and steroid sparing agents (azathioprine and mycophenolate mofetil) (3/21, 14.3%). About 28.6% (6/21) patients had a complete response, 61.9% (13/21) had a partial response, and 9.5% (2/21) had no clinical response. Patients with Tourette's syndrome and with isolated psychosis did not receive immunotherapies. Patients with psychotic symptoms received antipsychotics and mood stabilisers.

Median time from onset of neurological symptoms to first immunotherapy was 35 days (mean 525, range 2–2,920; data available in 9/21 patients with BGE-AE).

#### Outcome

In the BGE-AE subgroup, at a median follow-up of 5 years (mean 6.6, range 0.4–18, data available in 19/21) relapses occurred in 42.3% (9/21). At follow-up, 38.1% (8/21) had residual movement disorders (mostly mild dystonia), 42.3% (9/21) developed psychiatric disorders (particularly anxiety disorder), and 61.9% (13/21) had cognitive symptoms. Median mRS at last follow-up was 2 (mean 1.5, range 0–5, data available in 21/21). Data on outcome was not available in patients with Tourette's syndrome. In the psychosis subgroup, 2/3 were unable to return to school or start employment.

### Anti-GABAAR Autoimmunity

#### Demographics

Twenty-three children with anti-GABAAR autoimmunity were identified (20, 34–42) (13/23, 56.5% female). Median age at onset was 11 years (mean 9.6, range 2 months – 17 years).

#### Clinical Syndromes and Symptoms

AE was the most frequent clinical syndrome (18/23, 78.2%), followed by stiff-person syndrome (SPS), right temporal lobe epilepsy with hippocampal sclerosis, catatonia of unknown origin, West syndrome and Lennox-Gastaut syndrome, psychiatric disorder (1/23, 4.3% each). The most frequently described symptoms were seizures (21/23, 91.3%) cognitive impairment (9/23, 39%), behavioural change (5/23, 21.7%), movement disorders (39.1%, 9/23), and dysautonomia (17.4%, 4/23).

Hodgkin lymphoma was diagnosed in one patient, 10 months before neurological onset (1/23, 4.3%). About 17.4% of children (4/23) had viral infections before the onset of neurological symptoms: herpes labialis, HSV1 encephalitis, human herpes virus 6 (HHV6) encephalitis, and a concomitant parvovirus B19 encephalitis. One additional patient had fever without confirmed infection in the 6 days preceding neurological onset.

#### Anti-GABAAR Antibodies

Antibodies were tested in serum in 87% (20/23) patients (positive in 20/20, 100%), and in CSF in 69.6% (16/23) (positive in 12/16, 75%). 30.5% (7/23) patients had other coexistent autoantibodies: 4% (1/23) anti-GABABR antibodies (see also the anti-GABABR section), 13% (3/23) anti-NMDAR antibodies, and 13% (3/23) anti-GAD65 antibodies. CBA was used in 23/23 patients.

#### Investigations

Abnormal MRI was reported in 69.5% (16/23) patients, most commonly with increased T2/FLAIR signal in frontotemporal lobes (10/23, 43.5%), often multifocal and with cortical-subcortical involvement. In all cases with available data (17/23), EEG showed generalised slowing and/or epileptiform activity (17/17, 100%). CSF was abnormal in 66.7% (12/18) patients with available data.

#### Treatment

Overall, 78.2% (18/23) patients received first-line immunotherapy (IVMP and/or OP, IVIG, TPE), and 43.5% (11/23) received second-line immunotherapy and/or steroid sparers: rituximab (8/11), cyclophosphamide (2/11), and mycophenolate mofetil (1/11). Anti-seizure medications (ASM) were used in 52.2% (12/23) patients.

Median time from onset of neurological symptoms to first immunotherapy was 11 days (mean 28.8, range 2–91; data available in 4/23).

#### Outcome

At last follow-up (median 8 months, mean 13.3, range 2–36; data available in 12/23), relapses occurred in 53.8% (7/13) of patients with available data on disease course. Data on outcome was available in 19/23 patients. Median mRS at last follow-up was 4 (mean 2.4, range 0–4; data available in 5/19). Complete recovery was reported in 36.8% (7/19) patients, all treated with immunotherapy. Partial recovery was reported in 57.9% (11/19), and residual dysfunctions were cognitive deficits, need for chronic ASM, persistence of SPS symptoms. The death occurred in a 3-year-old child (1/19, 4%) with coexistent anti-GABABR antibodies, for sepsis during refractory status epilepticus (20).

### Anti-GlyR Autoimmunity

#### Demographic

Twenty-two children with anti-GlyR autoimmunity were identified (39, 43–53) (12/22, 54.5% female). The median age at onset was 9 years (mean 9.7, range 9 months −17 years).

#### Clinical Syndromes and Symptoms

Reported clinical syndromes were: mesial temporal lobe epilepsy with hippocampal sclerosis (MTLE-HS, 5/22, 22.7%), other epilepsy syndromes and epileptic encephalopathy (8/22, 36.4%), SPS (5/22, 22.7%), progressive encephalomyelitis with rigidity and myoclonus (PERM) (2/22, 9.1%), transverse myelitis (1/22, 4.5%), and acute disseminated encephalomyelitis associated with optic neuritis (1/22, 4.5%). Seizures (13/22, 59.1%), cognitive impairment (8/22, 36.4%), rigidity (7/22, 31.8%), myoclonus (7/22, 31.8%), and psychiatric disturbances (6/22, 27.3%) were the most frequent symptoms overall, followed by consciousness disturbances, dysautonomia, sleep disturbances, movement disorders, cranial nerve deficits, and brainstem signs. Seizures, cognitive impairment, and psychiatric symptoms were most represented in the MTLE-HS, epileptic encephalopathy, and epilepsy group, while rigidity and myoclonus were most frequent in patients with SPS and PERM.

No malignancies were reported. One patient “had a cold” 5 days before the neurological onset (47). About 13.6% (3/22) patients had other autoimmune diseases (one diabetes mellitus and two autoimmune thyroiditis).

#### Anti-GlyR Antibodies

Antibodies were tested in serum in 100% (22/22) of patients (positive in 18/22, 81.8%), and in CSF in 31.8% (7/22) (positive in 6/7, 85.7%). About 22/22 were tested with CBA.

#### Investigations

When available, abnormal brain MRI was reported in 46.2% (6/13, including all five cases of MTLE-HS reporting hippocampal sclerosis), abnormal EEG in 76.9% of patients (10/13, almost all cases in the MTLE-HS, epilepsy, and epileptic encephalopathy groups), and abnormal CSF in 11% (1/9).

#### Treatment

About 65% (13/20) of patients with available treatment data received first-line immunotherapy: IVIG (10/20, 50%), corticosteroids (8/20, 40%: 3/20 IVMP, 1/20 OP, and 4/20 corticosteroids not otherwise specified), and TPE (5/20, 25%). Rituximab was used in 5% (1/20), azathioprine in 10% (2/10). About 35% (7/20) patients did not receive immunotherapy (all in the MTLE-HS and epilepsy group) but received ASM and/or epilepsy surgery.

Median time from onset of neurological symptoms to first immunotherapy was 45 days (mean 344, range 44–943; data available in 3/22).

#### Outcome

At median follow-up of 24 months (mean 18.6, range 3–31; data available in 13/22), relapses occurred in 13.6% (3/22). Median mRS at last follow-up was 1 (mean 1.2, range 0–3; data available in 9/22).

### Anti-GABABR Autoimmunity

#### Demographics

Six children with anti-GABABR autoimmunity were identified (20, 54–58) (3/6 female). The median age at onset was 16 years (mean 13.6, range 3–18; data available in 6/6).

#### Clinical Syndromes and Symptoms

About 5/6 patients had limbic encephalitis and 1/6 AE. Limbic symptoms were described in all (6/6): memory deficits, psychomotor agitation, declining mental status, and personality changes; 4/6 had seizures. No tumours were reported.

#### Anti-GABABR Antibodies

Antibodies were tested in serum in 6/6 patients (positive in 5/6), and in CSF in 4/6 (positive in 4/4). The antibody assay used was CBA in 5/6; information was not available in 1/6 (54).

#### Investigations

When available, abnormal brain MRI was reported in 2/3 cases (mostly T2/FLAIR hyperintensity of basal ganglia, but also with cerebellum and brainstem involvement), abnormal EEG in 1/2, and abnormal CSF in 2/2 cases.

#### Treatment

Overall 4/5 patients with available treatment data received first-line immunotherapy: corticosteroids (3/5), IVIG (3/5), and TPE (2/5).

#### Outcome

Median follow-up was 12 months (mean 11, range 5–18; data available in 5/6). No relapses occurred (0/5). Patients (3/4) treated with first-line immunotherapy had a good response, while as described in anti-GABAAR section a 3-year-old patient with concomitant CSF anti-GABABR and anti-GABAAR antibodies died. One patient (1/5) did not receive immunotherapy and had a spontaneous recovery. Median mRS at last follow-up was 2.5 (mean 2.5, range 1–4; data available in 2/6).

### Anti-AMPAR Autoimmunity

#### Demographics

Four children with anti-AMPAR autoimmunity were identified (59–63) (1/4 female). Median age at onset was 12 years (mean 11, range 2–18; data available in 4/4).

#### Clinical Syndromes and Symptoms

Patients (4/4) had encephalopathy with cognitive-behavioural disfunction (i.e., memory impairment, apathy, psychotic symptoms, hallucinations, bipolar disorder) and movement disorders.

No tumours were reported. Patients (2/4) had prodromal fever before onset of neurological symptoms.

#### Anti-AMPAR Antibodies

Antibodies were detected in serum in 3/4, and in CSF in 2/3 of patients tested. CBA was used in 3/4 patients, while type of antibody assay was not available in 1/4 (62).

#### Investigations

Abnormal brain MRI was reported in 1/4 cases, presenting T2/FLAIR hyperintensities with contrast enhancement in bilateral cerebellar hemispheres. EEG was abnormal in 2/4. CSF was abnormal in 1/3 cases, showing lymphocytic pleocytosis.

#### Treatment

Patients (3/4) received immunotherapy (corticosteroids and IVIG in 2/4, rituximab in 1/4). One patient received antipsychotics. Median time from onset of neurological symptoms to first immunotherapy was 83 days (mean 591.5, range 10–2,190; data available in 4/4).

#### Outcome

At last follow-up (median 36 months, mean 36, range 24–48; data available in 2/4), 2/4 of patients had full recovery while the remaining had progressive improvement. Relapses occurred in 1/3 of patients with available data on disease course. mRS was available in one patient only (mRS 4 at symptoms onset, mRS 0 at 48-month follow-up).

### Anti-Amphiphysin Autoimmunity

#### Demographics

Four children with anti-amphiphysin autoimmunity were identified (64, 65) (1/4 female). The median age at onset was 12 years (mean 11.5, range 9–12; data available in 4/4).

#### Clinical Syndromes and Symptoms

Patients (4/4) had limbic encephalitis with fever, encephalopathy, memory impairment, and refractory temporal seizures.

No associated tumours were described. Preceding upper respiratory infection was reported in 2/4.

#### Anti-Amphiphysin Antibodies

Antibodies were positive in serum in 4/4 (CSF not tested). One patient (1/4) was also positive to anti-GAD antibodies. Patients (3/4) were tested with ELISA, whereas type of antibody assay was not available in 1/4 (65).

#### Investigations

Abnormal brain MRI was reported in 4/4 patients, showing abnormal FLAIR signal in mediotemporal areas or T2 temporal lobes hyperintensity. EEG data was available in only one patient, showing multifocal bilateral epileptiform discharges. Abnormal CSF was reported in 2/4 of patients.

#### Treatment

Overall 3/4 received immunotherapy (IVMP in 2/4; OP in 1/4; IVIG in 2/4), and had a partial response. Median time from onset of neurological symptoms to first immunotherapy was 61 days (mean 1,237.3, range 1–3,650; data available in 3/4).

#### Outcome

At a median follow-up of 9 years (mean 4.5 years, range 6 weeks – 11 years; data available in 4/4), no relapses occurred. All patients showed a chronic course with refractory epilepsy (4/4), and neuropsychiatric or cognitive symptoms (3/4); 2/4 developed a severe disability, precluding the possibility to live independently, whereas the other 2/4 had a mild disability. Median mRS at last follow-up was 3 (mean 3, range 2–4; data available in 4/4).

### Anti-mGluR5 Autoimmunity

#### Demographics

Four children with anti-mGluR5 autoimmunity were identified (66) (2/4 female). Median age at onset was 15 years (mean 13, range 6–16 years; data available in 4/4).

#### Clinical Syndromes and Symptoms

Patients (4/4) had AE, with a decreased level of consciousness (3/4), seizures (3/4), and psychiatric disturbances (3/4) as most described symptoms.

3/4 patients had Hodgkin lymphoma; neurologic symptoms preceded tumour diagnosis.

#### Anti-mGluR5 Antibodies

Antibodies were tested in serum in 2/4 patients (positive in 2/2). Antibodies were tested in CSF in 4/4 patients (positive in 4/4). Immunochemistry and CBA were used in 4/4 patients.

#### Investigations

Brain MRI was abnormal in 2/4 of patients, showing frontal and occipital lobes and cerebellum involvement. EEG showed diffuse slowing of rhythms (3/4) and epileptiform discharges (2/4). CSF analysis showed pleocytosis (median 38 white blood cells, range 21–114; data available in 4/4) and oligoclonal bands (3/4).

#### Treatment

Patients (3/4) received immunotherapy: corticosteroids in 3/4, IVIG in 2/4, TPE in 1/4, rituximab in 1/4. Time from onset of neurological symptoms to first immunotherapy was not available.

#### Outcome

At last follow-up (median 33.5 months, mean 37.7, range 12–79; data available in 4/4) 2/4 of patients showed partial response while the remaining recovered completely. Relapse was documented in one patient presenting neurological symptoms in association with tumour relapse.

### Anti-mGluR1 Autoimmunity

#### Demographics

Two children with anti-mGluR1 autoimmunity were identified (67, 68) (2/2 males); age at onset was 3 and 6 years, respectively.

#### Clinical Syndromes and Symptoms

Both patients had acute cerebellar syndrome; one had unsteady gait and mild behavioural changes, the other unsteady gait, dysarthria, intentional tremor, and movement disorder (choreiform movements of the face and jerky movements of the fingers).

No associated tumours were identified. One patient had prodromal headache, fever, nausea, and vomit, and had a previous history of streptococcal pharyngitis.

#### Anti-mGluR1 Antibodies

Antibodies were detected in CSF (2/2). Only one patient was tested in serum (negative). CBA was used in 2/2 patients.

#### Investigations

In one patient mild cerebellar oedema at brain MRI was reported. Data on EEG was not available. The CSF analysis showed pleocytosis and oligoclonal bands in both patients.

#### Treatment

Both patients received IVMP and IVIG. The time from onset of neurological symptoms to first immunotherapy was 10 and 21 days.

#### Outcome

At the last follow-up (2.5 and 7 months, respectively), both patients had a rapid full recovery. mRS was available in one patient only (mRS 1).

### Anti-DPPX Autoimmunity

A 15-year-old male with anti-DPPX autoimmunity was identified (69), tested with indirect immunofluorescence CBA (Table 1).

### Anti-IgLON5 Autoimmunity

A 2-year-old boy with anti-IgLON5 autoimmunity was identified (70), tested with CBA (Table 1).

### Anti-Neurexin-3alpha Autoimmunity

No paediatric cases were identified.

## Discussion

We have carried out a systematic literature review on rare paediatric NSAS (D2R, GABAAR, GlyR, GABABR, AMPAR, amphiphysin, mGluR5, mGluR1, DPPX, IgLON 5, and neurexin-3alpha autoimmunity) (Tables 1, 2, Supplementary Table 1), excluding NSAS that are already well-characterised in children (NMDAR, LGI1, and CASPR2 autoimmunity) and MOGAD. Amphiphysin, which is actually an intracellular synaptic vesicle protein involved in vesicle recycling at presynaptic nerve endings, was included since this epitope could be exposed to antibodies during synaptic vesicle fusion and reuptake, enabling the antibody binding process and its subsequent entrance in the cell (71).

**Table 2A:** Diagnostic clues in rare NSAS in children, based on our literature review in paediatric age (antibodies targeting D2R, GABAAR, GlyR, GABABR, AMPAR, amphiphysin, mGluR5, mGluR1, DPPX, IgLON5, and neurexin-3alpha) and on previous literature data.

**Diagnostic clues in rare neuronal surface antibody syndromes (NSAS) in children**		
**Diagnostic clues**		Antibody target*
Overall clinical syndrome	Autoimmune / Limbic encephalitis	**GABAAR, GABABR, AMPAR**, **amphiphysin, mGluR5**, D2R
	Basal ganglia encephalitis, Tourette's syndrome, Sydenham's chorea, PANS/PANDAS	**D2R**
	Isolated epilepsy or epileptic encephalopathy	**GlyR**, GABAAR
	Stiff person syndrome (SPS)	**GlyR**, GABAAR
	Progressive encephalomyelitis with rigidity and myoclonus (PERM)	**GlyR, DPPX**
	Acute cerebellar syndrome	**mGluR1**
Clinical symptoms	Epileptic seizures	**GABAAR**, **GlyR**, **GABABR, amphiphysin, mGluR5**, D2R
	Drug-resistant epilepsy, status epilepticus, epilepsia partialis continua	**GABAAR**, **GlyR**, amphiphysin, mGluR5
	Temporal lobe epilepsy with hippocampal sclerosis	**GlyR** GABAAR
	Cognitive decline	D2R, GlyR, GABAAR, GABABR, amphiphysin
	Movement disorder	**D2R**, IgLON5, AMPAR, GlyR, **GABAAR**, GABABR, mGluR1
	Psychiatric disturbances	**D2R**, GlyR, GABAAR, GABABR, amphiphysin, mGluR5
	Rigidity, myoclonus	**GlyR, DPPX**
	Sleep disturbances	**IgLON5**, GlyR, D2R
	Dysautonomias	**GlyR, GABAAR**, mGluR1
	Cerebellar symptoms	**mGluR1**, D2R
	Poor outcome, poor response to immunotherapy	**Amphiphysin**
MRI features	Basal ganglia abnormalities	**D2R**, GABABR
	Increased T2/FLAIR signal in frontotemporal lobes, multifocal, cortical-subcortical involvement	**GABAAR**
	Hippocampal sclerosis	**GlyR, GABAAR**
	Cerebellum abnormalities	mGluR1, GABABR, GABAAR, AMPAR, mGluR5

*AMPAR, α-amino-3-hydroxy-5-methyl-4-isoxazolepropionic acid receptor; D2R, dopamine-2 receptor; DPPX, dipeptidyl-peptidase-like protein-6; GABAAR and GABABR, γ-aminobutyric acid-A and B receptor; GlyR, glycine receptor; IgLON5, immunoglobulin-like cell adhesion molecule 5; mGluR1 and mGluR5, metabotropic glutamate receptor type 1 and 5; NMDAR, N-methyl-D-aspartate receptor; NSAb, neuronal surface antibodies; PERM, progressive encephalomyelitis with rigidity and myoclonus; SCLC, small cell lung cancer; SE, status epilepticus; SPS, stiff-person syndrome*.

^*^Bolded antibody targets indicate higher relevance for the diagnostic clue indicated.

We have identified 94 published children with these rare NSAS and available individual patient data, the most frequent being anti-D2R (28/94), anti-GABAAR (23/94), and anti-GlyR (22/94) autoimmunity.

NSAb detection rate was generally higher in serum than in CSF, with rare exceptions (GABABR, GlyR, and mGluR1); whenever possible, both serum and CSF should be tested (72, 73). NSAbs bind to conformational extracellular epitopes of cell surface proteins, and their detection depends on methods that preserve the three-dimensional structure of the antigen. Therefore, CBAs with live or fixed eukaryotic cells to express the protein at the cell surface in its “physiological” state are the most used assays for NSAbs (72–74), and live CBAs are the gold standard in MOGAD (75). Commercial CBAs are generally used in clinical practice but antibody detection is improved by pairing with in-house diagnostics (immunohistochemistry, live CBAs, and neuronal cultures), although this may be challenging to implement in laboratories that lack the required expertise, and is therefore not always available (73, 76–79). Moreover, commercial CBAs do not cover all NSAbs (i.e., GABAR, GlyR, D2R, and neurexin-3alpha), posing an additional challenge in the identification of these rare NSAS.

Overall, AE was the most frequent clinical syndrome (57/94, 61%), including limbic encephalitis (GABABR, AMPAR, amphiphysin, mGluR5), BGE (D2R), and other AE (GABAAR, D2R). Although, other less common syndromes have variably been reported, such as isolated epilepsy/epileptic encephalopathy (15/94, 16%; GABAAR, GlyR), isolated psychiatric disorders (D2R, GABAAR), Tourette's syndrome, Sydenham's chorea, PANS/PANDAS (D2R), SPS (GlyR, GABAAR), PERM (GlyR, DPPX), acute cerebellar syndrome (mGluR1), and sleep and movement disorders (IgLON5) (Table 2A).

While several paediatric NSAS features are similar to the adult population, some important differences could be highlighted (Table 2B), the most striking being the rarer paraneoplastic aetiology in children (5/94, 5.3% of the whole cohort) (1–5), especially in anti-amphiphysin autoimmunity (where >80% of adult have an associated malignancy vs. 0/4 of our included pediatric patients), but except for anti-mGluR5 syndromes (associated with Hodgkin lymphoma in 3/4 children identified in our review). In our literature cohort, one additional patient with anti-GABAAR encephalitis had Hodgkin lymphoma and one with anti-IgLON5 autoimmunity was affected by Langerhans cell histiocytosis. The type of associated tumour may also differ from adults. Despite the overall rarer paraneoplastic aetiology in children, oncologic searches remain mandatory, also considering that neurological symptoms may precede tumour diagnosis (i.e., GABAAR, mGluR5) (66).

**Table 2B:** Rare NSAS (with antibodies targeting D2R, GABAAR, GlyR, GABABR, AMPAR, amphiphysin, mGluR5, mGluR1, DPPX, IgLON5, and neurexin-3alpha): main distinctive features in paediatric age compared to adults, as regards demographics, clinical features and association with tumour, based on our systematic literature review and on previous literature observations (3, 4, 23, 36, 66–68).

**Main distinctive features in paediatric age compared to adults in rare neuronal surface antibody syndromes (NSAS)**		
**NSAbs**	**Demographics and clinical features**	**Tumour**
D2R	Published patients are mostly in paediatric age (23)	-
GABAAR	30–50% of published patients are in paediatric age The possible association between dyskinesias and dysautonomias can suggest the diagnosis of anti-NMDAR encephalitis. Paediatric anti-GABAAR autoimmunity may develop after viral encephalitis (HSV1, HHV6, parvovirus B19) and coexist with anti-NMDAR antibodies. Compared with adults, children are more likely to have generalised seizures (36)	In children, lower association with tumour Tumour in adults: 27–40% (mostly thymoma, then non-Hodgkin lymphoma, SCLC)
GlyR	In children, the most frequent clinical presentation is with isolated epileptic syndromes and epileptic encephalopathy (in adults: SPS and PERM)	In children, no association with tumour Tumour in adults: 20% (thymoma, B-cell lymphoma, breast cancer)
GABABR^∧^	-	In children, no association with tumour^∧∧^ Tumour in adults: 50% (SCLC)
AMPAR^∧^	In children, no predominance of female gender as in adults^∧∧^	In children, no association with tumour^∧∧^ Tumour in adults: 60% (SCLC, thymoma, breast cancer)
Amphiphysin^∧^	In children, the main clinical syndrome is limbic encephalitis (in adults: SPS and PERM)^∧∧^	In children, no association with tumour^∧∧^ Tumour in adults: >80% (SCLC, breast cancer, melanoma)
mGluR5^∧^	In children, movement disorders included dystonic postures and oculogyric crisis (in adults: postural tremor or myoclonic jerks). Compared with adults, children had only generalized seizures and were more prone to develop SE and to show diffuse EEG slowing (66).	In children, frequent association with tumour (Hodgkin lymphoma)^∧∧^ Tumour in adults: 60% (Hodgkin lymphoma)
mGluR1^∧^	In the two paediatric patient included in our literature review, more acute onset of cerebellar symptoms in comparison to subacute onset in adults (67, 68)^∧∧∧^	In children, no association with tumour^∧∧∧^ Tumour in adults: 10–20% (Hodgkin lymphoma, T-cell lymphoma, prostate)
DPPX^∧^	In the single paediatric case included in our literature review, no prodromal severe gastrointestinal symptoms as described in adults	In children, no association with tumour^∧∧∧^ Tumour in adults: rare (B cell neoplasms)
IgLON5^∧^	-	In children, association with Langerhans cell histiocytosis^∧∧∧^ Tumour in adults: 0–10%
Neurexin-3α	No paediatric cases identified	Tumour in adults: unknown

*AMPAR, α-amino-3-hydroxy-5-methyl-4-isoxazolepropionic acid receptor; D2R, dopamine-2 receptor; DPPX, dipeptidyl-peptidase-like protein-6; GABAAR and GABABR, γ-aminobutyric acid-A and B receptor; GlyR, glycine receptor; IgLON5, immunoglobulin-like cell adhesion molecule 5; mGluR1 and mGluR5, metabotropic glutamate receptor type 1 and 5; NMDAR, N-methyl-D-aspartate receptor; NSAb, neuronal surface antibodies; PERM, progressive encephalomyelitis with rigidity and myoclonus; SCLC, small cell lung cancer; SE, status epilepticus; SPS, stiff-person syndrome*.

*^∧^Very rare in paediatric age (see also Table 1)*.

*^∧∧^Observation based on 4–6 paediatric patients only*.

*^∧∧∧^Observation based on 1–2 paediatric patients only*.

As previously observed in other NSAS (28, 80, 81), in our paediatric literature cohort a history of preceding viral encephalitis was relatively frequent (i.e., D2R, GABAAR) (28, 36), and overall there was less marked gender association than in adults (i.e., no female preponderance in AMPAR encephalitis).

Regarding clinical features, while SPS and PERM are the most frequent syndromes in adult anti-GlyR and anti-amphiphysin autoimmunity, in children isolated epileptic syndromes and limbic encephalitis appear predominant, respectively. In anti-GABAAR autoimmunity, children are more likely to have generalised seizures and a different type of movement disorder compared with adults; a subset of children has coexistent anti-NMDAR antibodies (36). Finally, in paediatric anti-mGluR1 autoimmunity, cerebellar ataxia appears to have a more acute onset of symptoms than in adults (67, 68).

The reason for the rarity of these NSAS overall and especially in children is not clear, possibly including their recent description, the general lower frequency of tumour in paediatric age (therefore decreasing one known potential trigger), and hypothetical age-dependent variations in the expression pattern of some neuronal surface antigens in childhood. Underdiagnosis is also possible, in view of their yet incomplete clinical characterisation and the above-mentioned diagnostic challenges, these latter also reflected by the considerable treatment delay observed in out literature cohort.

The effect of treatment timing and strategy on the final outcome is yet to be clarified in larger cohorts, as observed for anti-NMDAR encephalitis (82).

## Limitations and Conclusion

Our literature review is strongly affected by its retrospective nature and the low number of patients, limiting the possibility of drawing definite conclusions. Moreover, we acknowledge that not all patients included in our literature review were diagnosed *via* CBA.

Despite these limitations, to our knowledge this is the first systematic review focusing on rare paediatric NSAS, and may therefore contribute to their characterisation. This review discloses antibody-specific features in children, helping clinicians suspect NSAS. We suggest examining both serum and CSF with CBA for a broad NSAbs panel in children presenting with new-onset focal or diffuse neurological deficits, cognitive difficulties, psychiatric symptoms, seizures, and/or movement disorder of unknown origin, even in the absence of definite MRI, EEG, or CSF abnormalities. Larger cohorts are warranted to elucidate the clinical features of these rare NSAS in children, their paraclinical findings and the most effective treatment strategies.

## Author Contributions

CA, VM, MT, LT, AL, and CL: carried out the literature review and drafted the paper. MN, SS, and IT: supervised the literature review and contributed to the critical revision of the manuscript. All authors contributed to the article and approved the submitted version.

## Conflict of Interest

The authors declare that the research was conducted in the absence of any commercial or financial relationships that could be construed as a potential conflict of interest.

## Publisher's Note

All claims expressed in this article are solely those of the authors and do not necessarily represent those of their affiliated organizations, or those of the publisher, the editors and the reviewers. Any product that may be evaluated in this article, or claim that may be made by its manufacturer, is not guaranteed or endorsed by the publisher.

## Abbreviations

AE: autoimmune encephalitis
BGE: basal ganglia encephalitis
NSAbs: neuronal surface antibodies
NSAS: neuronal surface antibody syndromes.
