# Supplementary material for: Autoimmune Encephalitis and Other Neurological Syndromes With Rare Neuronal Surface Antibodies in Children: A Systematic Literature Review
Source: Front Pediatr. 2022 Apr 20;10:866074. doi: 10.3389/fped.2022.866074 (PMC9067304; doi:10.3389/fped.2022.866074)
Supplement: Supplementary file 1 [file Table_1.DOCX]

**Supplementary Table 1**

Supplementary Table 1. Systematic literature review on autoimmune encephalitis and other neurological syndromes with rare neuronal surface antibodies in paediatric age: data on the 94 published children identified with antibodies targeting D2R, GABAAR, GlyR, GABABR, AMPAR, amphiphysin, mGluR5, mGluR1, DPPX, IgLON5, neurexin-3-alpha.

| **Sex**  **Age at symptoms onset**  **(Reference in the main text)** | **Prodromal symptoms** | | **Main clinical syndromes (specific clinical symptoms)**  **mRS score at peak** | | **Tumour**  **Preceding infections** | **CSF** | **Brain MRI** | **EEG** | **Time to diagnosis/immunotherapy after clinical presentation**  **Immunotherapy**  **Other treatments** | **Duration of follow-up from disease onset**  **Relapsing/monophasic disease**  **Outcome (mRS; SARA score)** | **Antibodies (serum/CSF)** | **Testing**  **Method** |
| --- | --- | --- | --- | --- | --- | --- | --- | --- | --- | --- | --- | --- |
| **D2R (n=28)** |  | |  | |  |  |  |  |  |  |  |  |
| #1  F, 1.6 y  Dale et al., 2012 (23) | Fever | | Basal ganglia encephalitis (chorea, agitation, sleep disorder, loss of deambulation). mRS NA | | Infection | (Pathological in 9/12 not specified patients) | Normal | NA | NA/NA  IVMP, CS, IVIG | 1.5 years; 1 relapse (1 months) with complete response; mRS NA | Anti-D2R  S: positive  CSF: (negative in 4/12 not specified patients) | CBA |
| #2  F, 3.5 y  Dale et al., 2012 (23) | Fever | | Basal ganglia encephalitis (chorea, ocular flutter, personality change, cognitive decline, ataxia, tremors, regression of milestones). mRS NA | | None | (Pathological in 9/12 not specified patients) | Normal | NA | NA/NA  IVMP, CS, IVIG | 2 years; 2 relapses (3 and 12 months), with complete response; mRS NA | Anti-D2R  S: positive  CSF: (negative in 4/12 not specified patients) | CBA |
| #3  M, 3.5 y  Dale et al., 2012 (23) | None | | Basal ganglia encephalitis (dystonia, parkinsonism, encephalopathy, lethargy, slurred speech). mRS NA | | Infection | (Pathological in 9/12 not specified patients) | Basal ganglia T2 hyperintensity (putamen, caudate nucleus) | NA | NA/NA  No therapy | 8 years; residual coordination disorder, anxiety, mild cognitive disorder; mRS NA | Anti-D2R  S: positive  CSF: (negative in 4/12 not specified patients) | CBA |
| #4  M, 4 y  Dale et al., 2012 (23) | Fever | | Basal ganglia encephalitis (dystonia, dystonic tremor, encephalopathy, seizures). mRS NA | | Infection | (Pathological in 9/12 not specified patients) | Basal ganglia T2 hyperintensity (putamen, caudate nucleus, frontal cortex) | NA | NA/NA  IVMP, CS | 1 year; mRS NA | Anti-D2R  S: positive  CSF: (negative in 4/12 not specified patients) | CBA |
| #5  F, 5 y  Dale et al., 2012 (23) | Fever | | Basal ganglia encephalitis (parkinsonism, dystonia, pyramidal weakness, emotional lability, insomnia, confusion) , mRS NA | | None | (Pathological in 9/12 not specified patients) | Normal | NA | NA/NA  IVMP, CS | 5 years; residual hemidystonia, attention deficit disorder, oppositional defiant disorder, rigid behaviours; mRS NA | Anti-D2R  S: positive  CSF: (negative in 4/12 not specified patients) | CBA |
| #6  M, 5 y  Dale et al., 2012 (23) | None | | Basal ganglia encephalitis (parkinsonism, ophthalmoplegia, pyramidal weakness, somnolence), mRS NA | | Infection | (Pathological in 9/12 not specified patients) | Basal ganglia T2 hyperintensity (putamen, thalamus, midbrain, pons) | NA | NA/NA  No therapy | 10 years; mRS NA | Anti-D2R  S: positive  CSF: (negative in 4/12 not specified patients) | CBA |
| #7  F, 6 y  Dale et al., 2012 (23) | None | | Basal ganglia encephalitis (parkinsonism, lethargy, ataxia, cerebellar signs), mRS NA | | None | (Pathological in 9/12 not specified patients) | Basal ganglia T2 hyperintensity (putamen, caudate nucleus, globus pallidus) | NA | NA/NA  No therapy | 4 years: residual motor tics, attention deficit disorder, rigid thinking; mRS NA | Anti-D2R  S: positive  CSF: (negative in 4/12 not specified patients) | CBA |
| #8  F, 7 y  Dale et al., 2012 (23) | Fever | | Basal ganglia encephalitis (chorea, encephalopathy, seizures, aggression), mRS NA | | Infection | (Pathological in 9/12 not specified patients) | Basal ganglia T2 hyperintensity (putamen, caudate nucleus, globus pallidus, cerebral peduncles) | NA | NA/NA  IVMP, CS | 8 years; residual inattentive attention deficit disorder, impulsive, anxiety, mild cognitive disorder; mRS NA | Anti-D2R  S: positive  CSF: (negative in 4/12 not specified patients) | CBA |
| #9  F, 9 y  Dale et al., 2012 (23) | None | | Basal ganglia encephalitis (hemidystonia, hemichorea, oculogyric crises, ophthalmoplegia, hallucinations, disinhibition, compulsive touching, mutism, hiccough), mRS NA | | None | (Pathological in 9/12 not specified patients) | Basal ganglia T2 hyperintensity (putamen, caudate nucleus, globus pallidus, cerebral peduncles) | NA | NA/NA  IVMP, CS | 9 years; 1 relapse (8 years)  residual dystonia, attention deficit disorder, autistic spectrum disorder, obsessive-compulsive disorder, bipolar disorder, cognitive deficit; mRS NA | Anti-D2R  S: positive  CSF: (negative in 4/12 not specified patients) | CBA |
| #10  M, 14 y  Dale et al., 2012 (23) | Fever | | Basal ganglia encephalitis (dystonia, encephalopathy, agitation, cardiac arrhythmia), mRS NA | | Vaccine | (Pathological in 9/12 not specified patients) | Normal | NA | NA/NA  IVMP, CS | 5,5 years; residual dystonia, mild cognitive disorder; mRS NA | Anti-D2R  S: positive  CSF: (negative in 4/12 not specified patients) | CBA |
| #11  M, 15 y  Dale et al., 2012 (23) | None | | Basal ganglia encephalitis (orofacial dystonia, oculogyric crisis, psychosis, paranoia, agitation, compulsive touching, mutism, poor eye contact), mRS NA | | Infection | (Pathological in 9/12 not specified patients) | Normal | NA | NA/NA  IVMP, CS | 9 years; residual psychosis; mRS NA | Anti-D2R  S: positive  CSF: (negative in 4/12 not specified patients) | CBA |
| #12  M, 15 y  Dale et al., 2012 (23) | None | | Basal ganglia encephalitis (parkinsonism, oculogyric crisis, psychosis, paranoia, anxiety, agitation, somnolence, hiccough, pupillary dilatation), mRS NA | | Vaccine | (Pathological in 9/12 not specified patients) | Normal | NA | NA/NA  No therapy | 2 years; mRS NA | anti-D2R  S: positive  CSF: (negative in 4/12 not specified patients) | CBA |
| #13  M, 3.5 y  Pawela et al., 2016 (30) | None | | Basal ganglia encephalitis (status dystonicus, encephalopathy, dysarthria, then aphasia, agitation), mRS NA | | None | Normal | Basal ganglia T2 hyperintensity (caudate, putamen, globus pallidus) | Diffuse intermittent slowing in sleep | Right after clinical presentation  IVMP, CS  MMF (relapse) | 13 years; 1 relapse (7 years)  residual mild left hemidystonia, anxiety, impairing in attention, fine motor coordination, executive functions, memory; mRS NA | Anti-D2R  S: positive  CSF: NA | NA |
| #14  F, 5.5 y  Pawela et al., 2016 (30) | None | | Basal ganglia encephalitis (dystonia, akinesia, encephalopathy, perseverative speech), mRS NA | | None | Normal | Basal ganglia T2 hyperintensity (caudate) | Diffuse slowing | Right after clinical presentation  CS | 7 years; 1 relapse (6 years)  residual mild left foot dystonia, anxiety, impairing in attention, fine motor coordination, executive functions, memory, impulsivity, suicidal thoughts, self-harming behaviour, oppositional behaviour; mRS NA | Anti-D2R  S: positive  CSF: NA | NA |
| #15  F, 1,6 y  Pawela et al., 2016 (30) | None | | Basal ganglia encephalitis (dystonic tremor, clumsy gait, agitation, night terrors), mRS NA | | None | Normal | Basal ganglia T2 hyperintensity (caudate, putamen) | NA | NA/3 years  No therapy in the acute phase. CS, IVIG (when 4.5y) | 5.5 years; relapsing-remitting course from 18mo-4y  residual anxiety, impairing in attention, fine motor coordination, executive functions, memory; mRS NA | Anti-D2R  S: positive  CSF: NA | NA |
| #16  M, 5 y  Pawela et al., 2016 (30) | None | | Basal ganglia encephalitis (dystonia, akinesia, mutism, disinhibited behaviour), mRS NA | | None | NA | Basal ganglia T2 hyperintensity (caudate, putamen) | Posterior slowing | Within one week/within one week  IVMP, CS, IVIG | 3 years; residual anxiety, Tourette syndrome, obsessive-compulsive disorder, impairing in fine motor coordination and executive functions; mRS NA | Anti-D2R  S: positive  CSF: NA | NA |
| #17  F, 1 y  Mohammad et al., 2014 (28) | None | | Basal ganglia encephalitis (chorea, encephalopathy, feeding difficulty ) mRS NA | | Previous HSV encephalitis | Normal except for PCR HSV1+ at onset of HSV1 encephalitis  NA at relapse | Unilateral temporal cortical and white matter lesions on MRI | Left temporal lobe epileptic activity with slowing | NA/ 8 years  CS, IVIG  Other: antiviral drugs, ASMs, epilepsy surgery | 14 years; refractory epilepsy, intellectual disability, behavioral problems, developmental delay; mRS NA | Anti-D2R  S: positive  CSF: NA | CBA |
| #18  F, 0.6 y  Mohammad et al., 2014 (28) | None | | Basal ganglia encephalitis (chorea, encephalopathy), mRS NA | | Previous HSV encephalitis | Pleocytosis and IgG anti HSV1 at onset of HSV1 encephalitis  NA at relapse | Unilateral parietal cortical, and white matter, basal ganglia and thalamic lesions on MRI | NA | NA/3-4 weeks  IVMP, IVIG  Other: antiviral drugs, ASMs | 13 years; refractory epilepsy, dystonic quadriplegic cerebral palsy, intellectual disability; mRS NA | Anti-D2R  S: positive  CSF: NA  Anti-NMDAR  S: positive  CSF: NA | CBA |
| #19  F, 17 y  Dai et al., 2020 (32) | None | | Basal ganglia encephalitis (akinesia, walking unstably, fine tremor, behavioural abnormalities, reduced speech, hyporeflexia, impaired motor coordination), mRS NA | | None | Normal | Basal ganglia T1 hyperintensity and internal capsule hypointensity | Increased slow-wave present over the bilateral occipital and left frontotemporal regions | NA/9 months  IVMP, CS, IVIG,  RTX, MMF (relapse) | 5 months; 2 relapses (1 and 5 months); mRS NA | Anti-D2R  S: positive  CSF: positive | Elisa |
| #20  M, 1,5 y  Marques-Matos et al., 2018 (31) | Vomiting | | Basal ganglia encephalitis (sudden onset of altered mental status, dystonia, chorea, mutism, irritability, poor eye contact, unable to sit, axial hypotonia) mRS NA | | None | Normal | Basal ganglia T2 hyperintensity and bilateral restricted diffusion | Moderate encephalopathy with no epileptiform activity | NA/5 weeks  IVMP, CS, IVIG, AZA | 1.3 months; barely noticeable dystonic posturing of right leg and choreiform mandibular movements after intensive rehabilitation; mRS NA | Anti-D2R  S: negative  CSF: positive | CBA |
| #21  F, 13 y  Salamatova et al., 2022 (33) | None | | Autoimmune encephalitis (lethargy, cognitive decline, blunted personality, parkinsonism, dystonia), mRS NA | | Infection (influenza A) | Normal | Subtle T2 abnormalities in the periventricular white matter and parahippocampal gyri | Background slowing | NA/1 year  IVIG, TPE  Other: levodopa + deep brain stimulation | NA; residual parkinsonism (dyskinesia, disabling rigidity, bradykinesia, foot dystonia causing falls, dysarthria) with some improvement after DBS, however needing a wheelchair and an electronic communication device; mRS NA | Anti-D2R  S: positive  CSF: NA | NA |
| #22  M, 6 y  Dale et al., 2012 (23) | None | | Tourette’s syndrome, mRS NA | | Elevated (>400 UI/ml) antistreptolysin-O titer | NA | Normal | NA | 1 year/none  None | NA, mRS NA | Anti-D2R  S: positive  CSF: NA | CBA |
| #23  M, 6 y  Dale et al., 2012 (23) | None | | Tourette’s syndrome, mRS NA | | None | NA | Normal | NA | 1 year/none  None | NA, mRS NA | Anti-D2R  S: positive  CSF: NA | CBA |
| #24  F, 7 y  Dale et al., 2012 (23) | None | | Tourette’s syndrome, mRS NA | | None | NA | Normal | NA | 8 years/none  None | NA; relapsing and remitting severe course; mRS NA | Anti-D2R  S: positive  CSF: NA | CBA |
| #25  F, 4 y  Dale et al., 2012 (23) | None | | Tourette’s syndrome, mRS NA | | Streptococcal infection  elevated (>400 UI/ml) antistreptolysin-O titer | NA | Normal | NA | 4 years/none  None | NA, mRS NA | Anti-D2R  S: positive  CSF: NA | CBA |
| #26  M, 11 y  Pathmanandavel et al., 2015 (28) | None | | Psychosis (auditory and visual hallucinations), mRS NA | | None | NA | Normal | Normal | NA/NA  Other: antipsychotics | NA; good clinical response; mRS NA | Anti-D2R  S: positive  CSF: NA  Anti-NMDAR | CBA |
| #27  M, 15 y  Pathmanandavel et al., 2015 (29) | None | | Psychosis (auditory hallucinations, delusions, elevated mood), mRS NA | | None | NA | Normal | NA | NA/NA  Other: antipsychotics and mood stabilizer | NA; successful return to school but later relapses and unable to graduate high school; mRS NA | Anti-D2R  S: positive  CSF: NA | CBA |
| #28  F, 17 y  Pathmanandavel et al., 2015 (29) | None | | Psychosis (delusions, elevated mood), mRS NA | | None | NA | Normal | NA | NA/NA  Other: antipsychotics and mood stabilizer | NA; successfully graduated high school and started tertiary studies; mRS NA | Anti-D2R  S: positive  CSF: NA | CBA |
| **GABAAR (n=23)** |  | |  | |  |  |  |  |  |  |  |  |
| #1 F, 16 y  Petit-Pedrol et al., 2014 (20) | None | | Encephalitis (encephalopathy with cognitive and affective problems) with  tonic-clonic seizures  status epilepticus; mRS NA | | Hodgkin's  lymphoma | Pleocytosis  (23/ul) | Multifocal increased T2/FLAIR signal with cortical subcortical  involvement | Slow activity, bilateral  temporal and generalised periodic epileptiform discharges | NA/NA  IVMP, IVIG, TPE  RTX, CPH  Other: LEV, TPR  MDZ, Barbiturate | NA; mild cognitive deficit that is improving; mRS NA | Anti-GABAAR  S: > 1:1280  CSF: 1:320 | CBA |
| #2  M, 3 y  Petit-Pedrol et al., 2014 (20) | None | | Encephalitis (Confusion, lethargy, dystonia, chorea, opsoclonus, ataxia) with complex partial seizures and status epilepticus; mRS NA | | None | Pleocytosis (154/ul) and increased protein | T2/FLAIR hypersignal,  brainstem, cerebellum basal ganglia and hippocampus | Slow activity, bioccipital ictal activity | Died before diagnosis  IVMP, IVIG  Other: multiple ASMs, barbiturate coma, decompressive  craniectomy | Exitus for sepsis | Anti-GABAAR  S: NA  CSF: 1:320  Anti-GABABR | CBA |
| #3  M, 4 y  Petit-Pedrol et al., 2014 (20) | None | | Encephalitis with hemiparesis, followed by partial seizures and status epilepticus; mRS NA | | None | pleocytosis and increased protein | FLAIR changes  suggesting encephalitis | Slow activity with epileptiforn discharges | NA/NA  LEV | 36 mo; recovery, in antiepileptics therapy; mRS NA | Anti-GABAAR  S: 1:320  CSF: 1:40 | CBA |
| #4  M, 2 y  Petit-Pedrol et al., 2014 (20) | None | | Encephalitis with partial seizures, status epilepticus, choreoathetoid movements; mRS NA | | None | Normal | Cortical atrophy | Slow activity with right parietal ictal activity | NA/NA  CS  Other: CBZ, VPA, MDZ, LEV, ketogenic diet, barbiturate coma | 24 mo; cognitive and motor  skills improved, partial  seizures persist; mRS NA | Anti-GABAAR  S: 1:160  CSF: negative | CBA |
| #5  F, 15 y  Petit-Pedrol et al., 2014 (20) | None | | Encephalitis with seizures; mRS NA | | None | pleocytosis (8/ul) | Bilateral fronto- temporal  increased T2/FLAIR signal, leptomeningeal enhancement | Multifocal ictal activity | NA/NA  NA | NA | Anti-GABAAR  S: 1:160  CSF: NA  Anti-GAD | CBA |
| #6  F, 16 y  Petit-Pedrol et al., 2014 (20) | None | | Encephalitis with  orofacial dyskinesia, seizure and dysautonomia, behavioural changes, insomnia, decreased  level of consciousness; mRS NA | | None | pleocytosis (17/ul) | Left temporal cortical-  subcortical high T2/FLAIR signal | Slow activity | NA/NA  IVMP, IVIG, TPE, RTX  Other: VPA | NA; Full recovery; mRS 0 | Anti-GABAAR  S: 1:20  CSF: negative  Anti-NMDAR | CBA |
| #7  M, 12 y (5y)  Petit-Pedrol et al., 2014 (20) | None | | stiff-person syndrome  and episodic seizures; mRS NA | | None | NA | Hippocampal high  T2/FLAIR signal | Epileptiform discharges | NA/NA  IVIG, RTX  Other: LEV | NA; described mild improvement of SPS, free of seizures; mRS NA | Anti-GABAAR  S: 1:20  CSF: NA  Anti-GAD | CBA |
| #8  F, 11 y  Baysal-Kirac et al., 2016 (35) | None | | Right Temporal Lobe Epilepsy  in Mesial Temporal Lobe Epilepsy  and Hyppocampal Sclerosis (depression, psychotic spells, postoperative  hypersexuality.  Focal, Secondarily generalized Seizures,  Status Epilepticus  Aura: fear and pilomotor aura); mRS NA | | None | NA | Right hyppocampal sclerosis | Epileptiform discharges | NA/NA  Other: OXC with poor response, amygdalohippocampectomy | NA; (free of seizures or no more than a few early, non disabling seizures); mRS NA | Anti GABAAR  S: positive  CSF: NA | CBA |
| #9  M, 17 y  Pettingill et al., 2015 (34) | None | | Catatonia of unknown aetiology (intermittent drooling and long periods of  staring and verbigeration; at other times he was verbally unresponsive sat abnormally, with grimacing and posturing); mRS NA | | None | Normal | NA | Normal | 3 months/3 months  TPE (at onset and for relapse with good reponses), IVMP, IVIG; oral CS  Other: antidepressant antipsychotic and anxiolytic, no clinical response | 20 mo; 1 relapse (6 months); frontal dysfunction, reduced verbal fluency, severe apathy, emotional and social withdrawal, blunted affect, and difficulty in abstract thinking; mRS2 | Anti-GABAAR  S:  1:540 at onset  1:180 at relapse  CSF: negative | CBA |
| #10  F, 13 y  Pettingill et al., 2015 (34) | None | | Psychological disorder (Disorientation, behavioral  change, violence) and seizures (absences, nocturnal  generalized tonic clonic seizures); mRS NA | | Dysembryoplastic  Neuroepithelial tumor resected  earlier in life | NA | NA | NA | NA/NA  No immunotherapy  Other: ASMs and antipsychotics | 16 mo; NA | Anti-GABAAR  S: 1:540  CSF: NA | CBA |
| #11  F, 10 mo  Spatola et al., 2017 (36) | None | | Encephalopathy with status epilepticus (focal motor seizures, involuntary movements, decreased level of consciousness, coma, SE, autonomic instability), mRS 5 | | Yellow fever vaccine | Normal | Normal | Diffuse slowing, epileptiform  activity | NA/NA  CS, IVIG | 8 mo; partial recovery; mRS 4 | Anti-GABAAR  S: > 1:640  CSF: 1:40 | CBA |
| #12  M, 15 mo  Spatola et al., 2017 (36) | HSV1 encephalitis | | Encephalopathy with ataxia, coreoatetosis and status epilepticus, irritability, focal motor refractory seizures, dysphagia; mRS 5 | | HSV1 encephalitis 8 wk before | Pleocytosis  (53/ul) | New increased T2/FLAIR signal  in bilateral frontal and  temporal lobes | Generalized bilateral epileptiform activity | NA/NA  CS, TPE, RTX | 8 mo; partial recovery; mRS 4 | Anti GABAAR  S: 1:80  CSF: 1:120  Anti-NMDAR | CBA |
| #13  F, 14 y  Spatola et al., 2017 (36) | Headache, malaise | | Seizures, abnormal movements; mRS NA | | None | Pleocytosis  (51/ul) | Normal | NA | NA/NA  None | NA | Anti-GABAAR  S: 1:40  CSF: NA | CBA |
| #14  M, 16 y  Spatola et al., 2017 (36) | Headache, weight loss, vomiting | | Encephalopathy with seizures and dysautonomia, personality change, memory loss, insomnia, dysmetria and weakness, mRS 3 | | None | Pleocytosis (14/ul), oligoclonal bands | Bilateral temporal, frontal and occipital lobes increased T2,  flair signal  focal leptomeningeal gadolinium enhancement | Diffuse and focal slowing, bilateral periodic epileptiform discharges | NA/NA  CS, IVIG, TPE, RTX | 3 mo; mRS 0  NA; Complete recovery | Anti-GABAAR  S: 1:320  CSF: 1:80 | CBA |
| #15  F, 2.5 mo  Spatola et al., 2017 (36) | HHV 6 encephalitis 5 weeks before | | Encephalopathy with orofacial and generalized dyskinesias (seizures, hypoactivity, orofacial and generalized dyskinesias, decreased level of consciousness), mRS 5 | | HHV 6 encephalitis 5 weeks before | Pleocytosis (40/ul) and increased protein (0.85g/l) | Pathological but NA | Asymmetric slowing, bilateral epileptiform activity | NA/NA  CS, IVIG | 6 mo; partial recovery (only  seizures and dyskinesias  improved); mRS 4 | Anti-GABAAR  S: 1:160  CSF: 1:320  Anti-NMDAR | CBA |
| #16  M, 13 y  Spatola et al., 2017 (36) | History of focal seizures successfully treated with phenytoin | | Epilepsia partialis continua, episodes of focal motor seizures, secondary generalization, psychomotor agitation, mRS 2 | | None | Normal | Bilateral parietal and occipital lobes, right frontal lobe increased T2,  flair signal; some abnormalities persisted during periods free of symptoms | Asymmetric slowing (right). No epileptiform activity | NA/NA  CS, IVIG, RTX | 8 mo; complete recovery; mRS 0 | Anti-GABAAR  S: 1:1280  CSF: 1:320 | CBA |
| #17  F, 13 y  Caputo et al., 2018 (37) | Fever | | Encephalitis, FIRES like onset and super-refractory status epilepticus (focal, repeated, seizures mainly of mesial temporal and opercular bilateral.  Milder episodes with staring, unresponsiveness and chewing automatisms or arrhythmic clonic movements of the mouth and drooling); mRS NA | | Infection | Normal | Normal | Focal discharges lasting 3-10 minutes and involving alternatively the right and left temporal regions at 10-15 minute intervals | 8 days/2 days  IVIG, intravenous CS, TPE  Other: ASMs | 6 mo; 1 relapse (3 months) mild impairment in the working memory tests, anxiety and depressive traits; mRS 1 | Anti GABAAR  S: 1:200  CSF: 1:10 | CBA |
| #18  F, 10 y  Figlerowicz et al., 2017 (38) | Headache, fever, vomiting | | Autoimmune encephalitis (slurred speech, dysphagia, salivation and left-sided muscle weakness, epilepsy partialis continua, status epilepticus with focal motor seizure of the left lower limb, deficits in ability to learn, increased deep tendon reflexes); mRS NA | | Herpes labialis 2 wk earlier | Pleocytosis (20/ul) | Disseminated areas of hyperintensity in both hemispheres and multiple cortical-subcortical abnormalities with increased FLAIR and T2 signal involving mainly the left temporal, frontal, and parietal lobes | Periodic delta slow waves after sharp wave activity in the right medial temporal lobe | NA/NA  IVMP, IVIG, oral CS, TPE, RTX  Other: ASMs | NA; 3 relapses in 5 months then complete recovery; mRS 0 | Anti-GABAAR  S: 1:160  CSF: negative  Anti-GAD | CBA |
| #19  F, 2 mo  Tekturk et al., 2018 (39) | None | | West Syndrome and Lennox-  Gastaut Syndrome (severe motor mental delay, spasticity, myoclonus) | | None | NA | Normal | Background EEG activity: diffuse theta and delta waves  Hypsarrhytmia and suppression burst pattern, followed by generalized multifocal spike and waves, rhythmic delta activity | NA/NA  IVIG  Other: ASMs including ACTH with good response | NA  NA; some relapses | Anti-GABAAR  S: positive  CSF: NA | CBA |
| #20  F, 8 y  Nikolaus et al., 2018 (40) | Fever, fatigue and reduced appetite | | Encephalitis with catatonia (hyperpnoea, delayed pupillary reaction, ataxia,  apathy, stupor, mutism); mRS NA | | Enterovirus, meningitis when 4 years old | Pleocytosis (19/ul), oligoclonal bands | Bilateral, asymmetrical,  multifocal supra and infratentorial  (sub-) cortical (cingulate cortex, temporomesial, frontopolar and  inferior lateral orbital gyrus) and cerebellar lesions (in total 12 well-defined lesions of 3-20 mm diameter), with T2-hyperintensity but no contrast uptake. No signs vasculitis on cerebral MR-angiography | Normal | 7-14 days/7 days  IVMP, IVIG, TPE | 2 mo; full recovery; mRS 0 | Anti-GABAAR  S: positive  CSF: positive | CBA |
| #21  F, 1 y  O’Connor et al., 2019 (41) | NA | | Encephalitis with refractory status epilepticus, coma; mRS NA | | None | Normal | Generalized atrophy | NA | NA/NA  IVMP, IVIG | 24 mo; residual severe cognitive impairment,  seizures; mRS 4 | Anti-GABAAR  S: NA  CSF: positive | CBA |
| #22  M, 16 y  O’Connor et al., 2019 (41) | Nausea, vomiting, weight loss | | Encephalitis with seizures; mRS NA | | None | NA | Multifocal bilateral cerebral lesions, nonenhancing (frontal, temporal, occipital), | NA | NA/NA  IVMP, IVIG, TPE, RTX | NA; full recovery; mRS 0 | Anti-GABAAR  S: positive  CSF: NA | CBA |
| #23  M, 6 y  Valle et al., 2021 (42) | Headache and self-limited  focal impaired awareness  seizures | | Super-refractory status  epilepticus during PVB19 Infection (focal seizures characterized by left-sided facial twitching without impaired awareness, evolving to persistent seizures, hemibody hyperreflexia, cerebellar ataxia); mRS NA | | Infection (parvovirus B19) | Pleocytosis  (14/ul) | T2/FLAIR hyperintensity  and a mild expansion of the left cerebellar hemisphere, with  some contrast enhanced foci and an absence of restricted diffusion, suggestive of an inflammatory and/or infectious process, such  as acute cerebellitis. | Epileptiform discharges | 79 days/15 days  IVMP, cyclosporine, TPE, IVIG  Other: ASMs, acyclovir | 3 years; relapses; mRS 0 | Anti-GABAAR  S: NA  CSF: positive | NA |
| **GlyR (n=22)** |  | |  | |  |  |  |  |  |  |  |  |
| #1  F, 14 mo  Damasio et al., 2013 (47) | Cold 5 days before neurological symptoms onset | | PERM Syndrome (irritability, restless sleep, sudden episodes of axial hyperextension, rigidity, generalized myoclonus, laterocollis to the right, left hemifacial spasm, trismus, urinary retention). mRS 5 | | None | Normal | Normal | Frequent generalized myoclonus during all sleep phases, without cortical correlates | 1.5 months/1,5 months  CS, IVIG  Other: acyclovir, cephtriaxone, chlarytromicin | 24 months; mild clinical recurrences associated with an infection, mild head trauma, reduction of steroid, or increased interval between immunoglobulins; mRS 1 | Anti-GlyR  S: 1:200  CSF: 1:2 | CBA |
| #2  M, 17 y  Atmaca et al., 2017 (43) | None | | Epilepsy (generalized tonic-clonic seizures, first status epilepticus). | | None | Normal | Normal | Generalized paroxysmal sharp and slow waves and postictal slowing | 1.5 months/NA  No immunotherapy  Other: status epilepticus interrupted with LEV. | NA; no recurrences, in therapy with levetiracetam and zonisamide; mRS NA | Anti-GlyR  S: 1:640  CSF: NA | CBA |
| #3  F, 14 y  Carvajal-Gonzalez et al., 2014 (44) | None | | PERM Syndrome. mRS 5 | | None | NA | NA | NA | NA  CS, IVIG, TPE | >24 mo; mRS 1 | Anti-GlyR  S: 1:640  CSF: NA | CBA |
| #4  F, 5 y  Carvajal-Gonzalez et al., 2014 (44) | None | | Epileptic encephalopathy. mRS 5 | | None | NA | NA | NA | NA  CS, IVIG, TPE | >24 mo; mRS 3 | Anti-GlyR  S: 1:2560  CSF: NA | CBA |
| #5  F, 8 y  Carvajal-Gonzalez et al., 2014 (44) | None | | ADEM with optic neuritis. mRS 5 | | None | NA | NA | NA | NA  CS, IVIG | >24 mo; mRS 1 | Anti-GlyR  S: 1:320  CSF: NA | CBA |
| #6  M, 3 y  Chan et al., 2017 (45) | None | | Autoimmune encephalitis (seizures, movement disorder [chorea, dystonia, stereotypy] and behavioural derangement); mRS NA | | None | Normal | NA | Normal | 49 days/44 days  IVMP, IVIG  Other: LEV, benzhexol | NA | Anti-GlyR  S: 1:160  CSF: NA | CBA |
| #7  F, 7 y  Clardy et al., 2013 (46) | None | | Stiff-Man Syndrome, classic phenotype, with symptoms on low back and lower limbs. mRS 2 | | None | NA | NA | NA | 1 year/NA  IVIG, TPE  Other: diazepam, baclofen | 3 mo; mRS 1 | Anti-GlyR  S: positive  CSF: NA  Anti-GAD65 in serum | CBA |
| #8  M, 14 y  Clardy et al., 2013 (46) | None | | Stiff-Man Syndrome, variant stiff trunk. mRS 3 | | None | NA | NA | NA | 3 years/NA  TPE  Other: diazepam, baclofen, dantrolene, botulinum toxin | 7 mo; mRS 1 | Anti-GlyR  S: negative CSF: positive  Anti-GAD65 in serum | CBA |
| #9  F, 5 y  Clardy et al., 2013 (46) | None | | Stiff-Man Syndrome, classic phenotype, with symptoms on lower limbs, trunk, head and neck. mRS 3 | | None | NA | NA | NA | 46 years/NA  IVIG, AZA  Other: diazepam, baclofen | 24 mo; initial good response to IVIG with mRS 1 but then worsened with mRS 3 at final follow up; mRS NA | Anti-GlyR  S: negative CSF: positive  Anti-GAD65 in serum | CBA |
| #10  F, 5 y  Hacohen et al., 2013 (48) | None | | Explosive-onset epileptic encephalopathy; mRS 5 | | None | NA | NA | NA | NA/NA  NA | NA | Anti-GlyR  S: positive CSF: positive | CBA |
| #11  M, 15 y  Hacohen et al., 2014 (48) | None | | Transverse myelitis, with leg weakness and sensory loss; mRS NA | | None | NA | Short cervical cord lesion on spinal MRI. Additional deep white matter lesions seen on cranial imaging. | NA | NA/NA  NA | NA | Anti-GlyR  S: positive  CSF: NA | CBA |
| #12  F, 5 y  McKeon et al., 2013 (50) | None | | Stiff-Man Syndrome, classic phenotype, with toe walking, thoracolumbar spasms, pain and scoliosis, gait freezing; mRS 2 | | None | Normal | Normal | NA | 50 years/NA  IVIG, AZA | 24 mo; near normal after 1 y treatment, no symptoms, mildly exaggerated lumbar lordosis; mRS 1 | Anti-GlyR  S: negative  CSF: positive  Anti-GAD65 in serum and CSF | CBA |
| #13  M, 14 y  McKeon et al., 2013 (50) | None | | Stiff-Man Syndrome variant phenotype (thoracic and lumbar spine only), with thoracic-lumbar spasms, pain and scoliosis; mRS 3 | | None | Normal | Normal | NA | 3 years/NA  CS, TPE | 12 mo; substantial improvements in spams following plasma exchange, symptoms worsened with prednisone alone; mRS NA | Anti-GlyR  S: negative  CSF: positive  Anti-GAD65 in serum | CBA |
| #14  F, 17 y  Piquet et al., 2019 (51) | None | | Autoimmune epilepsy (status epilepticus) accompanied by psychiatric symptoms and suicide attempt; mRS NA | | None | Pleocytosis (11/ul), normal proteins, no oligoclonal bands | normal | High amplitude delta waves with superimposed fast activity suggestive of extreme delta brush pattern | NA/NA  CS (Unclear benefit during acute hospitalization), IVIG (Initial improvement with seizure control and cognition during acute hospitalization), RTX (Initial improvement with one dose during acute hospitalization with resolution of seizures for 5 months)  Other: Lacosamide, zonisamide, clobazam | 12 mo; relapse with recurrent seizures and psychiatric symptoms at 6 months after initial presentation, now maintained on chronic therapy and stable one year with controlled seizures on 3 ASMs; mRS NA | Anti-GlyR  S: positive  CSF: NA  Anti-GAD65 and P/Q type calcium channel antibody in serum | CBA |
| #15  M, 9 months  Tekturk et al., 2018 (39) | None | | Epileptic encephalopathy (Lennox-gastaut syndrome); mRS NA | | None | NA | NA | Background activity: paroxysmal theta and delta waves.  Interictal activity: Generalized and sometimes focal spikes over right fronto-temporal region.  Ictal EEG: NA | NA/NA  No immunotherapy  VPA, LEV, TPR, with poor response. | NA | Anti-GlyR  S: positive  CSF: NA | CBA |
| #16  F, 14 y  Tekturk et al., 2018 (39) | None | | Epileptic encephalopathy, with generalized tonic and tonic-clonic seizures; mRS NA | | None | NA | NA | Background activity: diffuse theta and delta waves.  Interictal activity: generalized multifocal spike and polyspike and slow waves.  Ictal EEG: generalized polyspikes | NA/NA  IVIG (good response)  Other: CBZ, VPA (with moderate response to ASMs) | NA | Anti-GlyR  S: positive  CSF: NA  Associated serum antibodies against uncharacterized membrane antigens of cultured live hippocampal neurons | CBA |
| #17  F, 16 y  Vanli-Yanuz et al., 2016 (52) | None | | Mesial temporal lobe epilepsy with hippocampal sclerosis (frontal lobe and verbal memory disorder, focal seizure with impairment of consciousness and focal seizure evolving to bilateral convulsive seizure); mRS NA | | None | NA | Left hippocampal sclerosis | Left fronto-temporal spikes | NA/NA  No immunotherapy  Other: OXC, TPR, left amygdalohippocampectomy. | NA | Anti-GlyR  S: positive  CSF: NA | CBA |
| #18  M, 17 y  Vanli-Yanuz et al., 2016 (52) | None | | Mesial temporal lobe epilepsy with hippocampal sclerosis (frontal lobe and verbal memory disorder, focal seizure with impairment of consciousness and focal seizure evolving to bilateral convulsive seizure); mRS NA | | None | NA | Bilateral hippocampal sclerosis and white matter lesions | Right temporal sharp waves, activation during hyperventilation | NA/NA  No immunotherapy  Other: CBZ | NA | Anti-GlyR  S: positive  CSF: NA | CBA |
| #19  M, 9 y  Vanli-Yanuz et al., 2016 (52) | None | | Mesial temporal lobe epilepsy with hippocampal sclerosis (focal seizure with impairment of consciousness and focal seizure evolving to bilateral convulsive seizure); mRS NA | | None | NA | Left hippocampal sclerosis | Non-specific slow waves | NA/NA  No immunotherapy  Other: CBZ, LEV | NA | Anti-GlyR  S: positive  CSF: NA | CBA |
| #20  F, 15 y  Vanli-Yanuz et al., 2016 (52) | None | | Mesial temporal lobe epilepsy with hippocampal sclerosis (frontal lobe and visual memory disorder, focal seizure with impairment of consciousness and focal seizure evolving to bilateral convulsive seizure); mRS NA | | None | NA | Right hippocampal sclerosis | 11 seizures from the right fronto-temporal area, 8 from the left fronto-temporal area | NA/NA  No immunotherapy.  Other: CBZ, LEV, TPR Right anterior temporal lobectomy. | NA | Anti-GlyR  S: positive  CSF: NA | CBA |
| #21  M, 9 y  Vanli-Yanuz et al., 2016 (52) | None | | Mesial temporal lobe epilepsy with hippocampal sclerosis (frontal lobe and verbal memory disorder, focal seizure with impairment of consciousness, dysthymia-obsessive personality); mRS NA | | None | NA | Right hippocampal sclerosis | 3 seizures from right fronto-temporal area, right fronto-temporal epileptic focus, Fp2 slowing | NA/NA  No immunotherapy.  Other: LTG. Right amygdalohippocampectomy. | NA | Anti-GlyR  S: positive  CSF: NA | CBA |
| #22  M, 34 mo  Wuerfel et al., 2014 (53) | None | | Drug-resistant focal epilepsy, episodic behavioral disturbances, headache, clumsiness, intermittently impaired speech.; mRS NA | | None | Normal | Normal | Interictal EEG: multifocal, predominantly frontal and right parietal irregular spike waves and polyspike waves. | 2 years and 7 months/ 2 years and 7 months  IVMP (good response)  Other: sulthiame, LTG (only temporary amelioration of seizures) | 9 mo | anti-GlyR  S: 1:400  CSF: negative | CBA |
| **GABABR (n=5)** |  | |  | |  |  |  |  |  |  |  |  |
| #1  M, 18 y  Chen et al., 2017 (54) | None | | Limbic encephalitis (memory impairment, indifference, agitation, declined mental status, disorientation, cognitive impairment, sleep disorders), generalised tonic-clonic seizures; mRS 1 | | None | Pleocytosis (10/uL); intrathecal IgG synthesis | Normal | Normal | 1 month//NA  IVIG | 5 mo; mild residual short-term memory deficit; mRS1 | Anti-GABABR  S: positive  CSF: positive | NA |
| #2  F, 16 y  Höftberger et al., 2013 (55) | None | | Limbic encephalitis (Memory impairment, personality changes); mRS NA | | None | NA | NA | NA | NA/NA  CS, IVIG, TPE | 18; complete recovery; mRS NA | Anti-GABABR  S: positive  CSF: NA | CBA |
| #3  F, 16 y  Jeffery et al., 2013 (56) | None | | Limbic encephalitis (Declined mental status, psychomotor agitation, seizures); mRS NA | | None | NA | NA | NA | NA/NA  CS, TPE | 14 mo; complete recovery; mRS0 | Anti-GABABR  S: 1:1920,  CSF: NA  Anti-VGCC (N-type) in serum | CBA |
| #4  F, 18 y  Jeffery et al., 2013 (56) | None | | Limbic encephalitis (Memory impairment, seizures); mRS NA | | None | NA | NA | NA | NA/NA    None, spontaneous recovery | 6 mo; complete recovery; mRS0 | Anti-GABABR  S: 1:1920  CSF 1:64  Anti-VGCC (N-type and P/Q type) in serum | CBA |
| #5  M, 11 y  Liu et al., 2021 (58) | None | | Encephalitis with aggressive behaviour, personality changes, fever; mRS 4 | | Infection (Japanese encephalitis 24 days before) | NA | Abnormal (hyperintense lesions on the T2/FLAIR images in the bilateral thalamus and basal ganglia) | NA | 24 days/NA  NA | 12 mo; emotional instability; mRS 1 | Anti-GABABR  S: negative  CSF: positive | CBA |
| #6  M, 3 y  Petit-Pedrol et al., 2014 (20) |  |  | |  |  |  |  |  |  |  |  |  |
| **AMPAR (n=4)** |  | |  | |  |  |  |  |  |  |  |  |
| #1  M, 2 y  Qiao et al., 2021 (59) | Fever before onset | | Epilepsy, apathy, slow response, unstable walking and lethargy; mRS NA | | None | Normal | Normal | Widespread abnormalities, frequent spikes and multiple spikes-slow waves | 1.4 weeks/1.4 weeks  IVMP, IVIG, LEV | 48 mo; complete recovery; mRS NA | Anti-AMPAR  S: positive  CSF: negative | CBA |
| #2  M, 10 y  Trung Hieu et al., 2021 (61) | Fever | | Multiple focal seizures, confusion, cognitive behavioral disorders (time disorientation, speech dysfunction, amnesia, hyperactivity disorder, irritability) insomnia and movement disorders; mRS NA | | None | Normal | Normal | Slowed background, multifocal spikes, and wave-like epileptic discharges | 5 months/2 weeks  IVMP, IVIG, OXC, TPR, LEV | NA; relapse at 5 mo; later he remains hyperactive and easily agitated, improved seizures; mRS NA | Anti-AMPAR  S: positive  CSF: positive | CBA |
| #3  M, 18 y  Laurido-Soto et al., 2019 (63) | None | | Abulia, ocular flutter, asymmetric appendicular and truncal ataxia; mRS NA | | None | Pleocytosis | T2/FLAIR hyperintensities with contrast enhancement in the bilateral cerebellar hemispheres | NA | 5 months/5months  IVMP, IVIG, RTX | 24 mo; complete recovery; mRS NA | Anti-AMPAR  S: negative  CSF: positive | CBA |
| #4  F, 14 y  Quaranta et al., 2015 (62) | NA | | Resistant continuous rapid cycles bipolar disorder, extrapyramidal disorders, cognitive impairment, memory loss; mRS NA | | None | NA | Normal | Normal | 6 years/6 years  Other: Antipsychotic treatment, memantine | NA; progressive improvement of psychopathological condition and global functioning; mRS NA | Anti-AMPAR  S: positive  CSF: NA | NA |
| **Amphipysin (n=4)** |  | |  | |  |  |  |  |  |  |  |  |
| #1  M, 12 y  Chou et al., 2013 (64) | None | | Limbic encephalitis (encephalopathy, memory impairment and intractable seizures). Respiratory failure, PICU. mRS NA | | Upper respiratory infection | Pleocytosis (5/ul) | Abnormality in the mediotemporal area in FLAIR | NA | 4 years/no treatment  No immunotherapy | 5 y; refractory epilepsy, severe cognitive impairment and disability; mRS NA | Anti-amphiphysin  S: positive  CSF: NA | Elisa |
| #2  F, 13 y  Chou et al., 2013 (64) | None | | Limbic encephalitis (encephalopathy, memory impairment and intractable seizures), mRS NA | | Upper respiratory infection | Proteins > 45 mg/dl | Abnormality in the mediotemporal area in FLAIR | NA | 10 years/10 years  OP | 11 y; refractory epilepsy and hallucinations, good response once immunotherapy was administered; mRS NA | Anti-amphiphysin  S: positive  CSF: NA  ANA in serum | Elisa |
| #3  M, 12 y  Chou et al., 2013 (64) | None | | Limbic encephalitis (encephalopathy, memory impairment and intractable seizures). Respiratory failure, PICU. mRS NA | | None | Normal | Abnormality in the mediotemporal area in FLAIR | NA | 2 months/ 2 months  IVMP, IVIG | 2 years; refractory epilepsy, severe cognitive impairment and disability, alterations of behaviour, dysphagia; mRS NA | Anti-amphiphysin  S: positive  CSF: NA  Anti-GAD and anti-Ma1 in serum | Elisa |
| #4  M, 9 y  Lin et al., 2011 (65) | Fever | | Limbic encephalitis (encephalopathy, memory impairment and intractable seizures), mRS NA | | None | Normal | T2 hyperintensity of temporal lobes | Multifocal epileptiform discharges over bilateral hemispheres | 1 day/1 day  IVMP, IVIG | 1,2 months; Mild cognitive impairment and motor disability; mRS NA | Anti-amphiphysin  S: positive  CSF: NA | NA |
| **mGluR5 (n=4)** |  | |  | |  |  |  |  |  |  |  |  |
| #1  M, 16 y  Spatola et al., 2018 (66) | Headache | | Limbic encephalitis (Decreased level of consciousness, behavior and psychiatric symptoms) dystonia, generalized seizures; mRS 4 | | HD (stage IIIB) | Pleocytosis (31/ul)  Oligoclonal bands | Normal | NA | NA/NA  CS, TPE  Other: chemotherapy | 48 months; Complete recovery;  After complete recovery neurological relapse followed by HD relapse; mRS 0 | Anti-mGluR5  S: >1:1280  CSF: 1:20 | CBA |
| #2  M, 6 y  Spatola et al., 2018 (66) | Rash, headache, flu-like symptoms | | Limbic encephalitis (Psychomotor slowness, decreased level of consciousness, memory impairment, sleep disturbances), aphasia, dystonia, oculogyric crisis, ataxia, speech and motor regressions, hypoventilation, status epilepticus; mRS NA | | None | Pleocytosis (21/ul) | Increased T2/FLAIR signal of bilateral frontal (L>R) and right occipital lobes, cerebellum | NA | NA/NA  CS, IVIG, RTX | 19 months; Partial recovery;  mRS 3 | Anti-mGluR5  S: NA  CSF: 1:10 | CBA |
| #3  M, 15 y  Spatola et al., 2018 (66) | Headache, nausea | | Limbic encephalitis (Confusion, psychiatric symptoms, hallucination, decreased verbal output, attention deficit), status epilepticus; mRS 5 | | HD (stage IIA) | Pleocytosis (114/ul)  Oligoclonal bands | Increased T2/FLAIR signal of bilateral (L>R) posterior cortical diffusion restriction | NA | NA/35 days  Other: Chemotherapy, radiotherapy | 72 months; complete recovery; mRS 0 | Anti-mGluR5  S: NA  CSF: positive | CBA |
| #4  M, 15 y  Spatola et al., 2018 (66) | None | | Limbic encephalitis (Altered behaviour, anxiety, irritability, visual hallucinations, insomnia), facial paralysis; mRS 4 | | HD (stage I) | Pleocytosis (45/ul)  Oligoclonal bands | Normal | NA | NA/NA  CS, IVIG  Other: chemotherapy | 12 mo; partial recovery;  residual memory deficits; mRS 2 | Anti-mGluR5  S: 1:1280  CSF: 1:640 | CBA |
| **mGluR1 (n=2)** |  | |  | |  |  |  |  |  |  |  |  |
| #1  M, 3 y  Bien et al., 2020 (67) | None | | Cerebellar syndrome  Unsteady gait and tendency to fall with progressive inability to walk unassisted, Mild behaviour change; mRS 4 | | None | Pleocytosis (28/ul)  Oligoclonal bands | NA | NA | 3 weeks/3 weeks  CS | 7 mo; almost complete recovery, mild gait disturbance; mRS 1 | Anti-mGluR1  S: negative  CSF: positive | CBA |
| #2  M, 6 y  Spatola et al., 2020 (68) | Headache, fever, nausea, vomits | | Cerebellar syndrome (unsteady gait, dysarthria, intentional tremor) and movement disorders (choreiform movements of the face, jerky movements of the fingers, mRS 4 | | Streptococcal pharyngitis (2 months before) | Pleocytosis (125/ul)  Oligoclonal bands | Mild cerebellar edema on day 12; MR spettroscopy showed decreased NAA/Cr-Ratio and increased lactate | NA | 10 days/10 days  CS, IVIG  Other: cephotaxime and Penicillin V | 2.5 mo; complete recovery; mRS NA | Anti-mGluR1  S: NA  CSF: positive | CBA |
| **DPPX (n=1)** |  | |  | |  |  |  |  |  |  |  |  |
| #1  M, 15 y  Balint et al., 2014 (69) | None | | PERM syndrome  (Hyperekplexia, ataxia, nystagmus, stiffness, hyperreflexia, cognitive impairment); SARA Score 11/40; mRS NA | | None | Pleocytosis (28/ul)  Intrathecal IgG and IgM synthesis | Normal | Normal | 1 year/1 year  CS, TPE, IVIG with poor response; relapse at 42 months treated with RTX with good response | 15 mo; paartial response in SARA score (6/40); mRS NA | Anti-DPPX  S: 1:10000  CSF: 1:320 | CBA |
| **IgLON5 (n=1)** |  | |  | |  |  |  |  |  |  |  |  |
| #1  M, 2 y  Ye et al., 2021 (70) | None | | Sleep disorders (noisy sleep and movements during sleep), horizontal nystagmus, unsteadiness in posture and bilateral extension and stiffness of ankles; mRS NA | | Langerhans cell histiocytosis | Normal | Enhancement of the meninges and spinal cord | Normal | 2 months/2 months  Immunotherapy | NA | Anti-IgLON5  S: 1:30  CSF: negative | CBA |

Legend: ACTH: adenocorticotropic hormone; AMPAR: α-amino-3-hydroxy-5-methyl-4-isoxazolepropionic acid receptor; GAD: glutamic acid decarboxylase; ASMs: anti-seizure medications; AZA: azathioprine; CBA: Cell Based Assay; CBZ: carbamazepine; CPH: cyclophosphamide, CS: corticosteroids; CSF: cerebrospinal fluid; D2R: dopamine-2 receptor; DPPX: dipeptidyl-peptidase-like protein-6; EEG: electroencephalography; F: female; GABAAR and GABABR: γ-aminobutyric acid-A and B receptor; GlyR: glycine receptor; HD: Hodgkin disease; IgLON5: immunoglobulin-like cell adhesion molecule 5; IVIG: intravenous immunoglobulin; IVMP: intravenous methylprednisolone; LEV: Levetiracetam; LTG: lamotrigine; MMF: mycophenolate mofetil; M: male; mo: month/months; mGluR1 and mGluR5: metabotropic glutamate receptor type 1 and 5; MDZ: midazolam; MRI: magnetic resonance imaging; mRS: modified Rankin Scale; NA: not available; OXC: oxcarbazepine; RTX: rituximab; S: serum; TPE: therapeutic plasma exchange; TPR: topiramate, y: year/years; VGCC: voltage gated calcium channel; VPA: valproate.
